# Disclosure decisions: the combined effects of reciprocity, comparisons, and question sequences

**DOI:** 10.3389/fpsyg.2026.1605386

**Published:** 2026-03-17

**Authors:** Christos Themistocleous, Anastasios Pagiaslis, Andrew Smith, Yaniv Hanoch

**Affiliations:** 1 Persuasive Tech Lab, Cyprus University of Technology, Limassol, Cyprus; 2 Marketing, Tourism and Analytics, University of Nottingham, Nottingham, United Kingdom; 3 N-Lab, University of Nottingham, Nottingham, United Kingdom; 4 Wolverhampton Business School, University of Wolverhampton, Wolverhampton, United Kingdom

**Keywords:** decision making, dyadic relationships, experiment, privacy, reciprocity, sequence effects, social proof

## Abstract

**Premise:**

The study examines how different information-collection tools influence individuals’ voluntary disclosures of sensitive, private information. The individual and combined effects of dyadic relationships comparisons, and question sequences were tested.

**Methods:**

A 3 × 3 × 3 between-subjects main experiment was utilized. Using 27 unique online data collection methods, 1,276 participants were randomly assigned to a condition to measure actual voluntary disclosure using a pre-tested set of 18 questions with varying levels of invasiveness, covering themes such as drug use, sexual preferences, medical conditions, and consumption choices.

**Results:**

Findings show that invasive questions asked later in the questionnaire maximize divulgence. Using statements to justify the purpose of the information request enhances disclosure percentages. Participants would also mimic the disclosure behavior (or abstention from it) of a majority. Combinations of these factors indicate that disclosure reciprocity is best established by having easy questions asked first. The use of a triple combination was better than the use of these factors individually, except for comparison-inducing messages.

**Discussion:**

Our findings expand our understanding of how reciprocity can be established in question-based settings (i.e., through chatbots, structured interviews, medical questionnaires, etc.). Implications emerge on aspects that elevate concerns, for example, by overwhelming individuals with information on comparisons and data use. We map interactions and propose future research to further isolate combinations of disclosure techniques as they are more reflective of real-life situations than their isolated examinations.

## 1 Introduction

Modern digital technologies have enabled the collection, storage, and management of large amounts of personal data. From information about patients’ history to consumers’ online consumption choices, the advantages of data use range from enhanced organizational and diagnostic decision-making to informed communication types that enhance persuasion. Consumer insights can further inform service development processes (Babina et al., 2025), produce psychographic profiles, and enhance personalized attention (Stremersch et al., 2025).

Individuals, however, are asked daily to navigate privacy concerns and balance them with customization. The ongoing need to strike a balance between data protection and data sharing can lead consumers to clam-up, avoiding divulgence of information that is to their benefit. These attitudes are further exacerbated by an increasing number of data breach scandals, ranging from Ashley Madison in 2015 to Petco in late 2025. As careful disclosures are of benefit to individuals on occasions such as medical diagnosis, social support, improved financial decisions, and tailored recommendations, insights into their drivers are important points of reflection for psychologists, behavioral economists, and practitioners.

Disclosure research, founded on Social Exchange Theory, identified consumers to be performing cost–benefit analyses prior to disclosure opportunities, with prominent models including *privacy calculus* (Dinev and Hart, 2006; Meier and Krämer, 2024). Privacy calculus posits that if the mental process generates a net plus result (benefits exceed costs), self-disclosure occurs. On the one hand, projected concerns from a disclosure include loss of privacy and embarrassment, and on the other hand, physical or psychological compensations such as relational depth, personalization, and monetary reward (Mikhaeil and James, 2023; Themistocleous et al., 2014).

Such cost–benefit models advanced our understanding of disclosure decisions, yet findings from behavioral sciences demonstrate that this mental balancing is *not* always accurate. For example, information asymmetry exacerbates uncertainty in cost–benefit analyses, thinning the line between careful and risky disclosures. Experimental findings from John et al. (2011) identified a majority of participants to divulge sensitive (and even incriminating) information to unprofessional-looking sites that prompted them to test “How bad they were,” compared to professional-looking ones of well-known institutions. This clam-up to well-known sites, and over-disclosure to risky ones, can position consumers in vulnerable situations, susceptible to unsolicited data use aiming at misinformation (D’Errico et al., 2024) or fraudulent exploitation (Balakrishnan et al., 2025). This challenges the systematic correctness of disclosure cost–benefit models like privacy calculus (Dinev and Hart, 2006), disclosure management (White, 2004) and other conceptual applications of Social Exchange Theory. Contextual effects in a risky environment can lead to misplaced trust, thereby reducing concerns that would have otherwise weighed on the costs side of the mental scale.

More gaps remain in relative parameters that tip the mental scale of prescribed models toward divulgence, and which the individual might underestimate their effects. For example, the impact of social comparisons is found to affect disclosure patterns greatly. Respondents who were informed of how a majority of others have answered a question divulged more information in subsequent questions within a questionnaire (Acquisti et al., 2012). Information that induces social comparisons appears to act as a push mechanism toward mimicking socially desirable behaviors. These examples can explain the privacy paradox where intentions to divulge are extensively mismatched with the actual disclosure decisions of individuals—disclosing sensitive information when their stated intentions signal strong avoidance, and vice versa (Barth and de Jong, 2017).

Another parameter that appears to covertly affect cost–benefit analyses relates to the order in which privacy-related questions are structured. Moon (2000) proposes that within a sequence of private questions, questions with low-invasiveness that precede invasive ones stimulate divulgence, albeit opposite results have also been recorded (Acquisti et al., 2012).

Finally, an important parameter often overlooked by traditional cost–benefit models is dyadic relationships (DRs). This relates to the requester offering a piece of information in return for some information from the participant, thus completing the reciprocity cycle. Research by Zimmer et al. (2010) focused on dyadic relationships and established reciprocity by providing a piece of information to the user before asking for a sensitive disclosure (i.e., “*WebMD collects gender information since many medical issues are gender specific. What is your gender?*”). The reasoned dyadic (RD) relationship, which explained how the information would be used if acquired, facilitated more disclosures among respondents than the condition that offered unrelated statements, as well as when no reciprocity was established.

The literature systematically points to a gap in the examination of parameters often overlooked by traditional cost–benefit models. Combinations of such parameters remain underexplored, and their investigations are encouraged (Moon, 2000; Zimmer et al., 2010). To our knowledge, no previous study has combined the three factors of question sequence (QS), Social Comparisons, and disclosure reciprocity through dyadic relationships (DRs) to assess synergistic effectiveness. Aiming to further our understanding of causal factors of voluntary disclosures, this study addresses the question: Do question sequence effects, social comparison messages, and dyadic relationships work well together, or are they better employed individually when facilitating voluntary disclosures? Through an empirical investigation, we seek to identify combinations of disclosure-facilitating factors that inform the design of information-collection systems, while theoretically bridging the three main streams of literature. We note that this research is interested in actual disclosures, not intentional disclosure, to increase ecological validity. Next, we review relevant research across the three literature streams of interest and establish the main hypotheses.

## 2 Theoretical background

### 2.1 Question sequence effects on disclosures

Question sequence effects refer to the presentation order of questions in a questionnaire and the effects that alternative question sequences might have on responses. Early investigations by the American Marketing Association identified that women’s attitudes on advertising were more favorable when questions about dresses preceded them than vice versa (see Bradburn and Mason, 1964). They explain that in the first example, initial questions about dresses provide the context for the answers, priming them for the advertising questions that follow. These implications prompted attempts to examine how question order can influence consumer satisfaction with public services, with Van De Walle and Van Ryzin (2011) demonstrating significant differences across order sequences. Another study found that sequencing questions to first ask individuals to list positive aspects of their job favorably influences their responses to the more general questions that follow (Bowling et al., 2009). The requirement to recall information on certain positive aspects in the first set of questions can serve as points of reference for individuals when addressing follow-up items, thereby creating directional effects (Kaplan et al., 2013; Şahin, 2021).

In the domain of privacy, findings on question sequences also show significant effects on divulgence. Acquisti et al. (2012) identified the descending (DES) question sequence (most intrusive questions asked first) to promote greater divulgence on sensitive and embarrassing questions than an ascending order. They propose that this sequence produces a foot-in-the-door effect, thus avoiding a clam-up. They explain that by asking the most invasive questions first, respondents feel less threatened by the lessening degree of privacy-invasiveness, while setting the tone for the survey’s level of privacy sensitivity.

Counterintuitive arguments in the literature regarding the relationship between question sequencing and disclosure are also present. Moon (2000), focusing on human–computer disclosures, argues that an ascending sequence (ASC) of privacy-invasive questions (easiest questions asked first, followed by difficult ones) facilitates information disclosure. The author explains that easy-to-answer questions early on warm up respondents, thus leading to greater information divulgence in subsequent questions. Subsequent research has adopted this view (Chandra et al., 2022; Zhang et al., 2024), in trying to increase perceived levels of humanness in discussions with chatbots to facilitate divulgence. Aligned with reciprocity, Zimmer et al. (2010) demonstrate that a slow, progressive approach of privacy-invasiveness through question order can better accommodate reciprocity. This line of research makes a case for easy-to-answer questions presented first in questionnaires, as this mimics interpersonal interactions in which questions with a high risk of offending are typically asked later in the discussion. We note that this reasoning also aligns with Social Penetration Theory and the fact that sensitive information is disclosed at the mature stages of a relationship (Altman and Dalmas, 1973).

### 2.2 Comparative nature (CN) and divulgence

Comparisons originate from our basic instinct of imitation and are attributable to our need for social acceptance. In decision-making, following social cues and relying on choices considered to be social defaults are perceived as options that contain less risk and are thus more preferable (Cialdini, 2021; McDonald and Crandall, 2015; Zalmanson et al., 2022). Frequent research applications demonstrate how social comparisons lead individuals toward behavioral adaptation that complies with those around them. Drawing on behavioral economic insights, social comparison nudges (Baumeister and Leary, 2017; Steffel et al., 2016) were employed, for example, in the United States for improving tax compliance rates. Individuals with outstanding tax balances received official letters informing them of the majority’s tax compliance. Procedurally, the social comparison messages disclosed certain information to the non-compliant individuals regarding the compliant behaviors of others, thus inducing comparisons and aiming at a specific behavioral response, that of mimicking the conformed majority.

Although social comparison messages are used in different contexts to influence different behaviors, we also note that adverse behavioral effects are present. Schultz et al. (2007) identify occasions where social comparisons can negatively impact energy consumption for consumers who consume significantly less energy than their neighbors. This comparative information created a negative spillover effect, leading individuals to adopt a more relaxed stance toward energy saving, attributable to moral licensing (Themistocleous and Karapanos, 2025). Comparative information that positions individuals above others in terms of energy-saving performance can create a sense of achievement toward this moral cause, consequently “licensing” a more relaxed stance toward it.

Within disclosure settings, herding effects were recorded in security behaviors for privacy. By showing participants the most popular security practices, participants tended to accept and mimic these healthy behaviors (Vedadi and Warkentin, 2020). Acquisti et al. (2012) further capitalized on the innate human tendency of comparison. They examined how statements, presented as histograms, reflecting how others answered sensitive questions (admitted to have engaged/not admitted to have engaged) would influence participants’ admission, subjected to these comparisons. Admittance and thus disclosure were higher when the individual was given a histogram of a majority that admitted to the same fact. They argue that admissions by others can reduce one’s perceived risks and, consequently, facilitate self-disclosure.

### 2.3 Disclosure reciprocity through dyadic relationships

Early research describes reciprocity as the mutuality of offering something back to someone who has provided you with something of value (Gouldner, 1960). Reciprocity serves as a major persuasion technique that can circumvent traditional cost–benefit analyses as proposed by respective models, aiming to retain mutuality between actors in an act or transaction. In disclosure settings, reciprocity indicates that people will disclose information in response to information previously revealed by another party.

Moon (2000) applied reciprocity in the process of self-disclosure, monitoring how social cues can influence user responses and treat computers as social actors. She further experimented with providing information before each question, about the computers’ configuration and speed, in an attempt to facilitate reciprocity. By providing information before a request for disclosure was made, she aimed to facilitate disclosure reciprocity. An example of the exchange is the following: “This computer has been configured to run at speeds up to 266 MHz. But 90% of computer users don’t use applications that require these speeds. So this computer rarely gets used to its full potential. What has been your biggest disappointment in life?” (p. 326). Results show the latter reciprocity-facilitating condition led to more self-disclosures than its absence (being presented only with questions).

More recent evidence echoes these findings and shows that chatbots using reciprocity triggers, such as providing useful information to the user (email addresses that would be of value to them), resulted in an increased probability that they would reciprocate by disclosing their email addresses (Adam and Benlian, 2024). This disclosure effect was significant compared to the absence of reciprocity. Zimmer et al. (2010) expanded on Moon’s (2000) study by delving deeper into different conditions of reciprocity examined through the concept of dyadic relationships, which refers to one-for-one information exchanges. They define dyadic conditions as the ability to build relationships between two parties involved in a survey by having the survey conductor provide a piece of information with every question asked to the respondent and request a piece of information in return. The objective was to facilitate reciprocity and to test, specifically, the reasoned dyadic condition for enhancing data collection systems.

Their dyadic condition tested whether the information offered justified the reasons for the asked question and whether this could facilitate more disclosures than an unreasoned dyadic (UNREA) condition or its absence (non-dyadic [NONDR]). An example of a reasoned dyadic condition would be “For optimal health, people should see a general practitioner at least once a year. How long has it been since you have seen a doctor?” (p. 405). The first part related to the reasoned statement, the latter to the request. Zimmer et al.’s results indicate that the reasoned condition has a stronger moderation of the relationship between disclosure intention and disclosure probability than the unreasoned and non-dyadic conditions. They explain that in the latter exchange, the information offered informs and prepares the respondent for the questions that follow by justifying the benefits and uses of disclosed data, alleviating concerns in the process. As such, a reasoned dyadic relationship not only establishes reciprocity but also aims at tackling information asymmetry by explaining to respondents how their data will be used.

### 2.4 Hypotheses

This study is designed to examine how combinations of the three disclosure-facilitating factors (question sequence, comparative nature, and dyadic relationship) favor voluntary disclosures. This approach aligns well with contemporary perspectives on online interaction, where users are simultaneously exposed to normative cues, comparison-based information, and attempts to establish reciprocity.

The first hypothesis re-examines the high-level conditions of each examined factor deductively. By high-level, we refer to conditions that were previously studied and found to yield the most disclosed information compared to their alternative conditions. Specifically, for the question sequence, we hypothesize that the ascending sequence of invasiveness serves as the high-level condition. We base this on two main premises. First, we consider Moon’s (2000) and Zimmer et al.’s (2010) proposition on how warm-up questions preceding invasive ones can prepare individuals for the more difficult disclosures that follow. Second, following Kaplan et al.’s (2013) and Bowling et al.’s (2009) reasoning, we argue that initial negative perceptions toward a survey with very invasive questions at the start (thus a higher chance of disclosure avoidance) can have a directional effect on the responses toward less invasive questions that follow. This can lead to consumer clam-up as disclosure avoidance for the initial invasive questions can manifest in the subsequent easy-to-answer questions.

For comparative purposes, we base the high-level condition on Acquisti et al.’s (2012) work. Specifically, we consider here the high comparative nature (HIGHCN) condition, which leads individuals to believe that a majority disclosed the requested information.

For dyadic relationships, we adopt Zimmer et al.’s (2010) view on reasoned dyadic statements. Their findings supported the use of acquired data to facilitate more disclosures than the unreasoned condition (unstructured reciprocity) or the non-dyadic condition (absence of reciprocity). As the flow of the conversation requires both parties to disclose relevant information on the topic with matching quality and quantity for reciprocity establishment (Altman and Dalmas, 1973), we note that the reasoned dyadic complies more fully with this principle. Formally, for the three high-level conditions, we hypothesize as follows:

*H1:* The ascending sequence (ASC), high comparative nature (HIGHCN), and reasoned dyadic (REA) conditions will result in more actual disclosures than the alternative conditions of each concept, respectively.

Next, we combine these factors to assess their synergistic effect on divulgence. A previous study has examined each of the three factors in isolation. Research proposes the value of triangulations for disclosure-facilitating concepts, acknowledging that a gap remains for empirical investigation (Zimmer et al., 2010; Moon, 2000). H2 seeks to address this gap. We hypothesize that the synergy among the three high-level conditions of each factor will yield more disclosures than the individual use of each high-level condition. Formally:

*H2:* Combining ascending sequence (ASC), high comparative nature (HIGHCN), and reasoned dyadic (REA) conditions, will result in more actual disclosures than their isolated use.

## 3 Research methods

To test our hypotheses, we conducted a 3 × 3 × 3 quasi-experiment. The three conditions for each of the three examined factors (question sequence, comparative nature, and dyadic relationship) served as the main points of interest, allowing us to identify how individual and combined effects favored against disclosures. The information items were presented in accordance with their respective conditions, resulting in 27 presentation techniques. Each was tested using a distinct online survey with the process explained below.

### 3.1 Development of privacy-related questions

To develop questions that capture respondents’ personal information, we drew on relevant literature (Acquisti et al., 2012; Zimmer et al., 2010) and sought input from three faculty members at a UK university to assess the diversity of themes. A list of 18 questions was drawn up with varying levels of sensitivity to create a more dynamic set of privacy-invasiveness. The themes ranged from consumption choices and minor embarrassing information for milder questions to sexual preferences and serious medical conditions for the more invasive ones. To ensure varying levels of invasiveness, we pre-tested the 18 items and measured the perceived level of privacy-invasiveness. This allowed us to structure question sequences by escalating or de-escalating invasiveness accordingly. A total of 122 UK-based participants served as our pre-test sample (*N* = 122; *m*_age_ = 32.68; *σ*_age_ = 10.81; 50% men and 50% women; and 75.41% Caucasian). A 10-point semantic differential scale (10 being very privacy-invasive) was used in alignment with the literature on optimum response categories (Preston and Colman, 2000). Amazon vouchers were used as incentives. This process allowed us to rank the 18 questions based on perceived invasiveness. Results of the 18 privacy-related items are summarized in Appendix I.

### 3.2 Conditions and questionnaire structure

**Figure 1:**
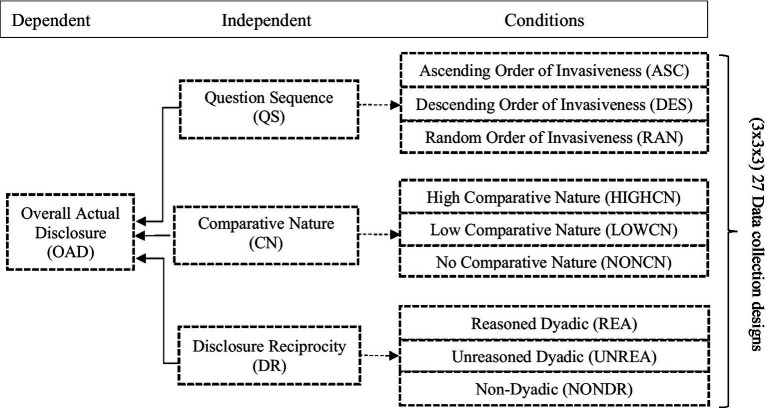
Synthesis of the 27 data collection designs.

Operationalization of the independent variables (question sequence, comparative nature, and dyadic relationship) involved the three conditions of each. Figure 1 provides a visual representation of the design and synthesis of the 27 conditions. Specifically, the question sequence (QS) utilized three conditions: the ascending (ASC) order condition presented the least invasive question at the beginning of the questionnaire. The descending (DES) order condition presented the most privacy-invasive questions at the start of the questionnaire (order as seen in Appendix I), and a random (RAN) order, which did not resemble any of the other two orders, ensuring that no apparent privacy-invasiveness (de)escalation was recorded, and thus a truly random sequence was in place.

For the comparative nature (CN), three conditions were employed, and each question was accompanied by disclosure patterns from a fictional pool of participants. This was used to trigger comparisons and test whether knowing about the patterns of others facilitated mimicking behavior for disclosures (Acquisti et al., 2012). In the high comparative nature condition (HIGHCN), participants were given messages informing them that the disclosure percentage of others was above the 90% disclosure mark (i.e., 96% of participants disclosed the following information; How many sexual partners have you had since you became sexually active?). The low comparative nature (LOWCN) condition offered disclosure percentages of others that were below the 45% mark (i.e., 38% disclosed the following information; How many sexual partners have you had since you became sexually active?). In the absence of a comparative nature [non-comparative nature (NONCN)], no information was provided regarding the response patterns of others. Appendix II summarizes the comparison-inducing percentages (how many people divulged and how many did not) in each respective condition.

Dyadic relationships (DRs) utilized three conditions. A total of 18 statements were presented to participants—one before each of the 18 questions. The reasoned dyadic (REA) condition provided statements that explained the direct reasons for requiring the asked information. For example, “The objective of this question is to assess how often you travel abroad and update you with offers and reduced travel prices. How many times a year you travel abroad either for holidays or business?” For the unreasoned (UNREA) condition, statements preceded each question, providing participants with information that had no direct relevance to the asked question and were used to assess the effectiveness of the unreasoned reciprocity pattern as proposed by Zimmer et al. (2010). For example, “Our company operates in 17 countries including the UK, Cyprus, Italy and Spain. How many times a year you travel abroad either for holidays or business?” Finally, for the non-dyadic relationship (NONDR), no information regarding the use of information was provided and disclosure reciprocity was absent. Appendix III summarizes the reciprocity statements provided in each condition.

### 3.3 Participants

UK participants were recruited via Prolific and compensated for their time at a rate of 8 pounds per hour. Controlling for demographic consistency between conditions and meeting respective quota requirements (i.e., age and gender), a total of 1,415 participants were recruited. Of the 1,415 participants, 139 responses were excluded due to incomplete or prolonged completion time. A final sample of 1,276 participants was used for our main analysis (*N* = 1,276; *m*_age_ = 26.73; *σ*_age_ = 8.85; 51.65% men and 48.35% women; and 86.71% Caucasian). Each respondent was randomly assigned to a single condition with an average of 47 participants per condition. Completion time was around 15 min. We conducted demographic consistency checks for age and gender to ensure that samples across conditions were similar and thus conditions were comparable. Analysis of variance (ANOVA) results for age demonstrated no significant differences between conditions; *F*(2,1,273) = 1.253, *p* = 0.178. A chi-squared test demonstrated no significant differences between conditions for gender; *χ*^2^(26) = 22.880, *p* = 0.640. Participants’ demographics and distribution for each condition are summarized in Table 1.

**Table 1:** Participant demographics per condition.

Condition	Participants	Men (%)	Women (%)	Age mean	Code
1	53	51	49	27.11	DES-NONCN-NONDR
2	43	56	44	26.98	DES-NONCN-UNREA
3	46	43	57	27.78	DES-NONCN-REA
4	65	45	55	25.20	DES-LOWCN-NONDR
5	44	73	27	26.41	DES-LOWCN-UNREA
6	54	57	43	26.15	DES-LOWCN-REA
7	49	59	41	27.92	DES-HIGHCN-NONDR
8	39	41	59	27.85	DES-HIGHCN-UNREA
9	44	45	55	26.41	DES-HIGHCN-REA
10	43	56	44	28.88	ASC-NONCN-NONDR
11	45	53	47	24.67	ASC-NONCN-UNREA
12	47	49	51	25.04	ASC-NONCN-REA
13	48	50	50	26.33	ASC-LOWCN-NONDR
14	48	54	46	24.83	ASC-LOWCN-UNREA
15	49	53	47	26.94	ASC-LOWCN-REA
16	45	49	51	27.09	ASC-HIGHCN-NONDR
17	59	59	41	27.93	ASC-HIGHCN-UNREA
18	47	49	51	24.23	ASC-HIGHCN-REA
19	37	57	43	29.11	RAN-NONCN-NONDR
20	54	56	44	28.22	RAN-NONCN-UNREA
21	41	51	49	25.34	RAN-NONCN-REA
22	40	38	62	24.78	RAN-LOWCN-NONDR
23	49	45	55	27.98	RAN-LOWCN-UNREA
24	47	51	49	27.91	RAN-LOWCN-REA
25	49	47	53	25.18	RAN-HIGHCN-NONDR
26	46	50	50	26.89	RAN-HIGHCN-UNREA
27	45	56	44	29.16	RAN-HIGHCN-REA
Total	1,276	51.6	48.4	26.73	

DES, descending order of privacy-invasiveness; ASC, ascending order of privacy-invasiveness; RAN, random order of privacy-invasiveness; HIGHCN, high comparative nature; LOWCN, low comparative nature; NONCN, non-comparative nature; REA, reasoned dyadic; UNREA, unreasoned dyadic; NONDR, non-dyadic.

### 3.4 Dependent variable

Once assigned to a condition, participants were asked to answer the 18 information items. A non-disclosure option was provided throughout the questionnaire to allow preference of avoidance and facilitate voluntary disclosures. We coded all responses as “1” for disclosure and “0” for non-disclosure, thus generating a percentage for each condition based on how many of the 18 questions were answered. This percentage [called overall actual disclosure (OAD)] was treated as the dependent variable in our study. To help mitigate inaccurate responses, we explicitly mentioned during the briefing the importance of truthful answers, reminding participants of making use of the “prefer not to disclose” option when feeling uneasy about a question, instead of falsifying information. Respondent anonymity was emphasized in the brief to mitigate social desirability bias. Although the intrusive and embarrassing questions are less frequent in organizational settings, the causal factors examined here influence decision-making about other, more general, and expectedly easier-to-answer privacy-related questions. We note that the use of sensitive questions to assess the impact of situational factors on divulgence is a widely used methodology in disclosure research settings (Acquisti et al., 2012; John et al., 2011; Moon, 2000; Norberg et al., 2007; Zimmer et al., 2010).

### 3.5 Procedure

To provide a clear context for recording answers, a scenario about a fictional data-capturing company was included in the study. The scenario indicated “*DataACC, an organization whose main activities revolve around the acquisition of personal information, asks you the following 18 questions.*” This was to mitigate an issue identified in the pilot study about the context and the entity that required their data. To enhance the study’s realistic nature, the scenario simulated a business setting, thus avoiding purposeful reduction of caution by respondents when addressing data-capturing questions (i.e., disclosures toward the organization of the principal investigator summarized in the first page). The questionnaire was designed in Qualtrics for online administration, enabling the randomization of items and making the administration of the 27 different conditions convenient. Both the pre-test and main study were granted approval by the Research Ethics Committee of a UK University [University of Nottingham].

## 4 Analyses

### 4.1 Hypothesis testing

For testing *H1*, we employed three one-way ANOVA test results where OAD served as the dependent variable, and each factor (QS, CN, and DR) served as the independent variable, respectively. *Post hoc* tests were accommodated each analysis, with results summarized in Table 2. For *H1* and question sequence, results were significant with *F*(2, 1,273) = 9.259, *p* = 0.000. *Post hoc* examinations identified the ascending order of privacy-invasiveness (easy-first-difficult-later) to be generating significantly higher OAD than the other two conditions of descending (difficult-first-easy-later) and random order, with the Tukey statistic being *p* < 0.001 and *p* < 0.05, respectively. No significant differences were found between the DES and RAN orders (*p* = 0.205). The latter indicates that a structured descending order, compared to an unstructured random order, generated statistically identical disclosures.

**Table 2:** ANOVA results.

Individual interactions							
Independent	Dependent	*F*	*p*	Condition	Mean (%)	Between groups	*Post-hoc* significance (Tukey HSD)
OAD	QS	9.259	<0.000^a^	ASC^d^	93.89	ASC × DES	0.000^a^
				DES	89.75	ASC × RAN	0.033^b^
				RAN	91.42	DES × RAN	0.205
OAD	CN	11.074	<0.000^a^	HIGHCN^d^	93.97	HIGHCN × LOWCN	0.000^a^
				LOWCN	89.43	HIGHCN × NONCN	0.066^c^
				NONCN	91.77	LOWCN × NONCN	0.043^b^
OAD	DR	3.775	<0.023^b^	REA^d^	93.25	REA × UNREA	0.042^b^
				UNREA	90.88	REA × NON	0.050^c^
				NONDR	91.95	UNREA × NON	0.997

a Significant at *p* < 0.001.

b Significant at *p* < 0.05.

c Significant at *p* < 0.10.

d Hypothesized high-level condition.

For comparative messages (CN), the ANOVA results were also significant [*F*(2, 1,273) = 11.074, *p* = 0.000]. The HIGHCN, which told participants that others disclosed at a high rate (i.e., 96% of others disclosed the following question) resulted in the highest OAD, demonstrating that statements that triggered comparisons led to significantly greater mimicking of others’ disclosure patterns. Here, comparative information offered *a priori* to each question behaves consistently with the patterns of ones offered *posteriori* as reported in Acquisti et al.’s (2012) experiments and expands the application of social comparison messages. Actual disclosure in the HIGHCN was significantly higher than the LOWCN (Tukey: *p* = 0.000) and the NONCN (Tukey: *p* = −0.066). Interestingly, LOWCN generated the lowest OAD even when compared to the NONCN (Tukey: *p* = 0.043), where comparative messages were absent, and no comparative information was provided. The latter denotes that participants mimicked the majority’s disclosure avoidance, demonstrating a two-way effect of comparisons in disclosure settings.

Dyadic relationships significantly influenced OAD [*F*(2, 1,273) = 3.775, *p* = 0.023]. *Post hoc* findings for the dyadic relationships examination showed that the Reasoned Dyadic, which provided information to the respondent on how the acquired data would be used, significantly increased disclosures compared to the Non-Dyadic (Tukey: *p* = 0.05) and the unreasoned dyadic (Tukey: *p* = 0.04) conditions. The unreasoned dyadic condition, which provided irrelevant pieces of information to participants, had no differences between the Non-Dyadic (Tukey: *p* = 0.997), indicating that irrelevant information to the asked question creates statistically similar disclosure effects as their complete absence. Findings here provide support for *H1*, identifying the ascending order, high comparative nature, and reasoned dyadic conditions as the high-level conditions for each of the examined factors, respectively (QS, CN, and DR).

For testing *H2*, we isolated the condition that combined all three high-levels, namely, ASC-HIGHCN-REA, and compared its overall actual disclosure with those generated by the three isolated conditions, namely, ASC, HIGHCN, and REA. The objective here was to examine whether a combination of the three high-level conditions worked better than *individually* applying them.

We conducted multiple comparisons using unpaired *t*-tests. Results are summarized in Table 3 under triple interactions. The high-level triple combination (ASC-HIGHCN-REA) generated significantly higher OAD than the isolated employment of ASC (*p* = 0.000; Cohen’s *d* = 0.456) and REA (*p* = 0.02; Cohen’s *d* = 0.472), respectively, but not with HIGHCN (*p* = 0.598; Cohen’s *d* = 0.109). This suggests that the triple combination should be preferred over the individual use of the ascending order and reasoned dyadic conditions. Nevertheless, the triple combination did not significantly outperform the isolated condition of high comparative nature. This means that combining social comparison messages with a question sequence and dyadic relationship improved actual disclosures only partially compared to when using them in isolation, thus providing only partial support for *H2*.

**Table 3:** Comparing triple and dual combinations.

Triple interactions										
Dependent	Condition	Mean (%)	SD	mean difference(*m*dif)	*t*	df	*p*	Cohen’s *d*	SE	95% CI
OAD	ASC–HIGHCN-REA^d^	96.34	5.59	4.22	2.192	88	0.000^a^	0.456	1.924	[0.397, 8.044]
	ASC^e^	92.12	11.83							
OAD	ASC–HIGHCN–REA	96.34	5.59	1.10	0.529	94	0.598	0.109	2.081	[−3.035, 5.230]
	HIGHCN^f^	95.24	13.18							
OAD	ASC–HIGHCN–REA	96.34	5.59	6.09	2.277	86	0.023^b^	0.472	2.679	[0.774, 11.426]
	REA^g^	90.24	17.38							

Dual interactions										
Dependent	Condition	Mean (%)	SD	*m*dif	*t*	df	*p*	Cohen’s *d*^h^	SE	95% CI^i^
OAD	ASC–REA	95.15	7.01	3.366	1.607	91	0.006^b^	0.332	2.095	[−0.794, 7.527]
	DES–REA	91.79	12.49							
OAD	ASC–REA	95.15	7.01	4.91	1.779	86	0.053^c^	0.371	2.760	[−0.577, 10.396]
	RAN–REA	90.24	17.38							

a Significant at *p* < 0.001.

b Significant at *p* < 0.05.

c Significant at *p* < 0.10.

d All three high-level conditions combined.

e Isolated ASC condition—CN absent and DR absent.

f Isolated HIGHCN condition—structured QS absent and DR absent.

g Isolated REA condition—structured QS absent and CN absent.

h Cohen’s *d* calculated using Hedge’s *g* modification as Cohen’s *d* = (*M* − *M*)/SDpooled, where SDpooled = (SD^2^ + SD^2^)/2. Cohen’s *d* was calculated using G*Power version 3.1.9.2.

i Bootstrapped results are based on 1,000 samples.

### 4.2 Additional analyses

Juxtaposed views by Zimmer et al. (2010) and Moon (2000) mention that disclosure reciprocity is best accommodated with an ascending question sequence. Our research design allowed us to test this claim. Using the condition that isolated the reasoned dyadic and ascending order (ASC-REA), we facilitated comparisons with the two conditions that used the reasoned dyadic but followed different question orders: descending order (DES-REA) and random order (RAN-REA). Our objective was to test certain configurations between different orders of questions and disclosure reciprocity. As listed in Table 3 (see dual interactions section), the results indicate that the ASC-REA generated more actual disclosures than the DES-REA (*p* = 0.006; Cohen’s *d* = 0.332) and RAN-REA condition (*p* = 0.05; Cohen’s *d* = 0.371). Specifically, the reasoned dyadic condition, which provides contextually relevant reciprocity, was better accommodated by a question sequence with slow escalation of invasiveness. This indicates that an ascending escalation of invasiveness allows disclosure reciprocity to take effect, most likely in parallel with the development of trust.

## 5 Discussion and implications

### 5.1 General discussion

The paper focused on certain contextual disclosure factors that are often overlooked by traditional cost–benefit models. We aimed to bridge attitude–behavior gaps of previous research by focusing on actual disclosures, as opposed to intended ones. We inform the conceptualization of future disclosure cost–benefit models and the design of information-collection processes that promote data-use transparency. Our examinations were comprised of two parts: Individual effect and combined effect examinations.

For the individual effects in question sequences, we identify that having mildly invasive questions asked first results in more disclosures than asking them at the end of the data collection process. Moon (2000) and Zimmer et al.’s (2010) propositions were verified through this examination, and the fact that the ascending invasiveness sequence can “loosen up” and prepare participants for the more difficult, privacy-invasive questions that are to follow. Acquisti et al.'s (2012) view, which favors the presentation of the most invasive questions at the start of the questionnaire, was not supported by our results. Findings here demonstrate that this descending approach leads to insignificant effects compared to a purely random order, making a case for a sequence that favors slow escalation of invasiveness. Results here can be linked to directional effects (Kaplan et al., 2013) and their impact on responses. We argue that difficult, negatively fused questions in terms of invasiveness can have negative directional effects on easier questions that succeed them. Avoidance of disclosure for the very invasive questions at the very beginning can manifest in the easier-to-answer questions that follow, ultimately increasing the chance for consumer clam-up for both invasive and non-invasive questions.

The second factor of interest was the comparative nature. Examinations confirm that participants respond to comparative stimuli when engaging in disclosures. Participants who were informed of how the majority behaved in terms of divulgence mimicked this behavior. A two-way, linear effect was recorded. If the majority disclosed the asked information, then the individual significantly favored disclosure; if the majority abstained from disclosure, then the individual would significantly refrain from divulgence. Our findings align with Loewenstein et al.’s (2014) claim on the effectiveness of social comparison messages, expanding them to show that disclosure abstention occurs when the majority refrains from disclosures.

The third point of focus was on dyadic relationships. Based on disclosure reciprocity (one-for-one; Cialdini, 2021), we identify that when providing a statement explaining the use of the requested information, it facilitates divulgence. Reciprocity that was established with unreasoned statements did not perform as well. This means that statements clarifying the use of data should be the go-to method for establishing reciprocity and thus divulgence. We attribute this to the alleviation of concerns due to transparency with how data will be used for an underlying personal or societal benefit. This works in conjunction with the principle of reciprocity (Zimmer et al., 2010). The unreasoned dyadic, which provided interesting but irrelevant information to the user, scored lower than the respective condition where it was absent. These statements might have been perceived as attempts to capture data by shifting the participant’s point of focus toward irrelevant information, thus intensifying their concerns. We link this finding to studies that provided compensation to reciprocate divulgence. They found that monetary returns for private information have been perceived as decoys, which elevated privacy concerns and led to abstention (Andrade et al., 2002).

For the combined effects, we examined dual and triple interactions. In dual combinations specifically, we combined the reasoned dyadic condition with each of the three question sequence types (ascending, descending, and random). The rationale was to examine which sequence best accommodated reciprocity and thus disclosures. Results find reciprocity to be working better when combined with the ascending order, empirically verifying Moon’s (2000) claim. We argue that easy questions asked first provide space for reciprocity to take its effect, as intimate questions asked too early could break down the reciprocity principle and result to clam-up. Structuring questions from easy to more difficult based on their invasiveness and complementing each question with explanations of how data will be used before asking for it, facilitates more disclosures compared to having the same explanations per question presented randomly or from most to least invasive questions.

For the triple interaction, we examined the combined effects of the three high-level conditions of reasoned dyadic, ascending sequence and high comparative nature. We constructed and compared this condition with how it favored against the individual employment of the conditions that comprised it. Our findings show a favorable synergistic behavior of the three conditions (ASC-HIGHCN-REA), resulting in more disclosures in the latter format than the individual one (REA). We argue that the ascending invasiveness sequence here accommodates better the reasoned disclosure reciprocity, allowing it to take its effect while comparative information appears to complement this interaction, ultimately generating more divulgence than the reasoned dyadic condition employed on its own.

When comparing the combined high-level condition (ASC-HIGHCN-REA) with the ascending sequence employed individually (ASC), results again favor the combination of the three for producing more divulgence. Both comparative messages and disclosure reciprocity reinforce the application of question sequences, thus demonstrating synergy toward disclosure maximization. Finally, comparing the three high-level conditions (ASC-HIGHCN-REA) with high comparative messages employed in isolation (HIGHCN), although the first generated more divulgence, the results were not significant (*p* = 0.598). Essentially, social comparison messages worked equally well on their own than when combined with disclosure reciprocity and question sequences. This highlights the effectiveness of social comparisons on divulgence, demonstrating strong effects that are not significantly improved by question order or reciprocity, although we note small effects in favor of the combined condition (*m*dif = 1.10).

### 5.2 Implications

Privacy discussions highlight consented data use and transparency of data-capturing processes as the main pillars of corporate digital responsibility (Lobschat et al., 2021) and sustainable marketing practices (Themistocleous, 2023). It is thus not surprising that disclosure behavior research shows particular interest in data collection methods that inform individuals and alleviate risks during disclosure deliberation. Our findings have direct implications for consumer research and organizational information accumulation.

First, for medical organizations where sensitive disclosures are essential to the precision of medical assessments, encouraging messages can help individuals engage in intimate disclosures that might otherwise evoke embarrassment or fear of stigma. Presenting them with how truthful disclosures benefited others can alleviate patients’ concerns. Our findings also point toward messages that inform respondents of a vast majority abstaining from disclosures, facilitating mimicking, and can reduce divulgence in a linear fashion. As such, search engines can provide tailored messages that promote disclosure avoidance of sensitive data to suspicious sites by displaying the number of visitors who declined disclosure requests. This is most prevalent on scam sites that target sensitive data such as credit card details and phone numbers.

Second, organizations can further utilize short, reasoned statements to establish reciprocity and explain the exact use and need for this data. This alleviates concerns and sets a reciprocal relationship for information exchange. Short, quick-to-read statements before each question are to complement more extended and complicated documents of organizational privacy policies. These points are amplified by Jensen et al.’s (2005) findings indicating that very few consumers read privacy statements (3%), while Turow et al. (2007), report that consumers believe that privacy policies in online settings imply data protection, while in reality, the opposite is true.

Third, in terms of questionnaire structure, a smooth sequential approach in terms of privacy-invasiveness (ascending sequence) needs to be prioritized. This sequence prepares individuals for the more difficult disclosures that are to follow. It also avoids negative directional effects and thus clam-up. Regarding information collection and data management, companies are advised to assess the invasiveness of questions before presenting them to respondents and accordingly structure them in an escalating order of invasiveness.

Fourth, when statements explaining data use are presented prior to each question, the sequence of the data collection process needs to be structured to present the easiest questions first and then progress to the more difficult ones. We identified the latter sequence to accommodate the establishment of reciprocity more effectively and thus provide more space for its development. This reduces the possibility for disclosure clam-up. Adding in the previous combination a third dimension through comparison-inducing messages can enhance divulgence further. Our triple combination examination, appears to maximize divulgence except when compared to high comparative nature which is only marginally lower. Directly informing individuals, as well as nudging them, through short explanations of data use can help with mitigating information asymmetries which can improve decisions at the time of the request (Acquisti et al., 2015).

### 5.3 Future research and conclusion

This research represents an expeditionary attempt to examine combined drivers of consumer disclosure behavior. The present design allowed for a partially inductive examination of combinations between the three factors of interest, opening new pathways for future research. Our investigation provides a map of factor interactions, yet further research can enhance the resolution of results by focusing on certain combinations. Follow-up examinations can focus on isolated synergies. Albeit more complex, synergies can help us map covert factors of divulgence for inclusion in future cost–benefit models. Context, for example, serves as an important parameter (John et al., 2011) and as such, dyadic relationships can be employed to explain data usage in both formal and informal settings and, respectively, assess their effects. Likewise, comparison-inducing messages can be employed in settings resembling scam sites and assess whether their effectiveness enhances disclosure decisions as it does in organizational settings.

Past experiences also serve as important proxies for future divulgence behavior. It is documented that privacy violations by organizations can have detrimental multi-level implications on trust and thus limit future disclosures (Tripathi and Mukhopadhyay, 2022). Examining how the presentation effects of data-capturing procedures can re-instill trust, especially when previous disclosure experiences of respondents have been negative, can further reinforce our understanding of the effectiveness of voluntary data-capturing processes as a trust-facilitating tool.

Finally, Westin’s (2003) consumer categorization in the sampling process could enhance findings relating to the effectiveness of data-capturing processes per group. Identifying respondents as either *fundamentalists*, *pragmatists* or *unconcerned* based on their intentions to disclose information can generate insightful findings. The examined techniques’ effectiveness will likely differ when targeting different types of consumers. For example, techniques that are effective on unconcerned individuals might not be as effective on fundamentalists. Although our research was interested in overall disclosure behavior and not the examination of specific polar views, we would expect that an individual’s predisposed propensity to disclose private information to an organization influences his/hers disclosure behavior. For example, a pragmatist is more likely to respond to social comparison messages when in deliberation, while fundamentalists (who have a strong predisposition toward avoidance) might not. Adding this categorization in future examinations can fuel further investigations into groups of consumers based on their propensity to voluntarily divulge information and the amount of influence exerted to them by each synergy during disclosure decisions.

## Data Availability

The raw data supporting the conclusions of this article will be made available by the authors, without undue reservation.
