# Supplementary material for: Disclosure decisions: the combined effects of reciprocity, comparisons, and question sequences
Source: Front Psychol. 2026 Mar 17;17:1605386. doi: 10.3389/fpsyg.2026.1605386 (PMC13036221; doi:10.3389/fpsyg.2026.1605386)
Supplement: Supplementary file 1 [file Supplementary_file_1.docx]

Appendix

Appendix I - Pre-test results: Rank from most to least privacy-invasive questions assessed by 122 participants

| Question Description | m | σ |
| --- | --- | --- |
| 1. How many sexual partners have you had since you became sexually active? | 7.920 | 2.286 |
| 2. What is the amount of your household savings? | 7.556 | 2.359 |
| 3. Have you ever been arrested? | 6.926 | 2.630 |
| 4. Have you ever looked at pornographic material? | 6.630 | 2.467 |
| 5. Have you ever tried illegal drugs like marijuana? | 6.482 | 2.578 |
| 6. Have you lied about your income to an official service? | 6.370 | 2.404 |
| 7. Have you ever suffered from cancer of any kind? | 5.963 | 3.192 |
| 8. Have you downloaded illegally obtained pirated songs and/or movies from the internet? | 5.778 | 1.340 |
| 9. Have you witnessed a serious crime and failed to report it or stop it? | 5.333 | 2.935 |
| 10. Have you called in sick when you were not sick either in your workplace, university, etc.? | 4.519 | 2.940 |
| 11. How much alcohol do you consume on average per week? | 4.074 | 2.745 |
| 12. Have you lied about your age to someone you were attracted to? | 3.926 | 2.448 |
| 13. Have you claimed to have education that you didn't actually have (either on your CV or in person) | 3.888 | 2.391 |
| 14. For which stores do you currently have loyalty cards? | 3.037 | 2.473 |
| 15. Have you knowingly wasted energy, for example by not switching off the lights for convenience at your workplace, school or university? | 2.444 | 2.486 |
| 16. Which mobile carrier do you currently use? | 2.440 | 1.826 |
| 17. How often do you travel abroad either for holidays or business? | 2.148 | 1.562 |
| 18. How many times do you go to a restaurant in an average week? | 1.741 | 0.903 |

**Appendix II *–* Percentages of how a majority responded to each question in each comparative condition**

| Question | High Comparative Nature | | Low Comparative Nature | | No Comparative Nature | |
| --- | --- | --- | --- | --- | --- | --- |
|  | Disclosed | Avoided Disclosure | Disclosed | Avoided Disclosure | Disclosed | Avoided Disclosure |
| 1. How many sexual partners have you had since you became sexually active? | 96% | 4% | 39% | 61% | - | - |
| 2. What is the amount of your household savings? | 89% | 11% | 43% | 57% | - | - |
| 3. Have you ever been arrested? | 91% | 9% | 38% | 62% | - | - |
| 4. Have you looked at pornographic material? | 90% | 10% | 37% | 63% | - | - |
| 5. Have you ever tried illegal drugs like marijuana? | 89% | 11% | 42% | 58% | - | - |
| 6. Have you lied about your income to an official service? | 92% | 8% | 40% | 60% | - | - |
| 7. Have you ever suffered from cancer of any kind? | 90% | 10% | 42% | 58% | - | - |
| 8. Have you downloaded illegally obtained pirated songs and/or movies from the internet? | 89% | 11% | 39% | 61% | - | - |
| 9. Have you witnessed a serious crime and failed to report it or stop it? | 88% | 12% | 43% | 57% | - | - |
| 10. Have you called in sick when you were not sick either in your workplace, university, etc.? | 93% | 7% | 36% | 64% | - | - |
| 11. How much alcohol do you consume on average per week? | 93% | 7% | 43% | 57% | - | - |
| 12. Have you lied about your age to someone you were attracted to? | 89% | 11% | 40% | 60% | - | - |
| 13. Have you claimed to have education that you didn’t actually have (either on your CV or in person) | 96% | 4% | 36% | 64% | - | - |
| 14. For which stores do you currently have loyalty cards? | 88% | 12% | 38% | 62% | - | - |
| 15. Have you knowingly wasted energy, for example by not switching off the lights for convenience at your workplace, school or university? | 88% | 12% | 42% | 58% | - | - |
| 16. Which mobile carrier do you currently use? | 90% | 10% | 43% | 57% | - | - |
| 17. How often do you travel abroad either for holidays or business? | 93% | 7% | 41% | 59% | - | - |
| 18. How many times do you go to a restaurant in an average week? | 92% | 8% | 40% | 60% | - | - |

Appendix III – Dyadic Relationship Statements

| Question | Reasoned Dyadic Relationship | Unreasoned Dyadic Relationship | Non-Dyadic |
| --- | --- | --- | --- |
| 1. How many sexual partners have you had since you became sexually active? | *The purpose of this question is strictly for confidential medical purposes and the promotion of HIV/AIDS examinations.* | The organisation employs 13000 individuals globally. Once a year all employees go through free medical checks including HIV and venereal diseases checks to ensure their health-related wellbeing. | **-** |
| 2. What is the amount of your household savings? | *This question is used for the profiling of respondents based on their income for advertising purposes.* | The organisation grossed 89.78 million British Pounds for the year 2012-2013. | ***-*** |
| 3. Have you ever been arrested? | *This question is asked in order to assess the overall number of people that have been arrested at least once in their life.* | DataACC offers equal opportunities for candidates that are interested in working at the organisation without any racial or gender discriminations. | **-** |
| 4. Have you ever looked at pornographic material? | *Here we are interested in capturing information for the profiling of how many individuals are introduced to pornographic imagery.* | A recent study funded by DataACC indicates that extensive use of pornographic imagery reduces the efforts of finding a long-term partner. | **-** |
| 5. Have you ever tried illegal drugs like marijuana? | *The objective of this question is to capture how many individuals tried at least once soft illegal drug for profiling purposes.* | In 2012 DataACC funded a project which examined the benefits and implications of soft drugs on the psychological health of UK adults. | ***-*** |
| 6. Have you lied about your income to an official service? | *The purpose of this question is to generate a percentage of the respondents who, for their own reasons, lied to an official service.* | DataACC has a revolving line of credit with well-known banks across Europe. | **-** |
| 7. Have you ever suffered from cancer of any kind? | *The purpose of this question is strictly for confidential medical profiling purposes and the optional provision of information by our organisation regarding help lines etc.* | *DataACC makes yearly donations to Queens Medical School in Nottingham for cancer research.* | **-** |
| 8. Have you downloaded illegally obtained pirated | *The objective of this question is to assess the preference of individuals when it comes to illegally downloading, or buying the original songs from online sources like iTunes etc.* | DataACC’s central information processing system was humorously named “Hal 9000” serving as a reference to Stanley Kubrick’s “2001:Space Odyssey”. | **-** |
| 9. Have you witnessed a serious crime and failed to report it or stop it. | *The objective of this question is to capture how many individuals have failed to report a serious crime that they witnessed.* | A recent survey indicates that individuals with high confidence help their colleagues more frequently. | **-** |
| 10. Have you called in sick when you were not sick either in your workplace, university, etc.? | *The reason for this question is to assess the overall tendency by individuals to excuse themselves from work for no valid reason, and accordingly design a campaign in order to reduce the number of individuals who engage in this activity.* | The average annual leave for the organisation’s employees is 28 days. | **-** |
| 11. How much alcohol do you consume on average per week? | *Here we are interested in profiling individuals according to their consumption of alcohol.* | DataACC was funded by Brent Hinds, born in Atlanta Georgia after finishing his second masters’ degree at the University of Nottingham. | ***-*** |
| 12. Have you lied about your age to someone you were attracted to? | *Information acquired from this question will be used as part of a research that studies human relationships.* | The overall average age of our employees is 37 years old. | **-** |
| 13. Have you claimed to have education that you didn’t actually have (either on your CV or in person) | *The reason for this question is to assess the overall tendency by individuals* *to claim to have education that they don’t have.* | The organisation’s current CEO is entrepreneur Anastasios Aristidopoulos. | ***-*** |
| 14. How many loyalty cards do you currently have? | *Here, the objective of this question is to assess the loyalty of consumers to multiple organizations.* | DataACC collaborates with several well known UK based grocery shops. | ***-*** |
| 15. Have you knowingly wasted energy, for example by not switching off the lights for convenience at your workplace, school or university? | *This question’s objective is to assess whether you unnecessarily waste energy and accordingly provide you with guidelines on how to end this habit and save money for yourself and others while preserving the environment.* | DataACC implemented recently the SAVE project in which all company cars are replaced by hybrids to preserve the environment. | ***-*** |
| 16. Which mobile carrier do you currently use? | *Here we are interested in identifying which mobile phone carrier you are using in order to provide you with relevant promotions from this carrier.* | A recent study showed that the UK mobile phone carrier market is one of the 10 largest markets in the country. | **-** |
| 17. How many times a year you travel abroad either for holidays or business? | *The objective of this question is to assess how often you travel abroad and update you with offers and reduced travel prices.* | *DataACC is a multi-national organisation operating in 17 countries including the UK, Greece, Luxemburg, Cyprus, Italy and Spain.* | **-** |
| 18. How many times do you go to a restaurant in an average week? | *This question is asked in order to assess the frequency of your visits to restaurant and provide you with new options of which restaurants you could enjoy.* | *The organisation is comprised by 56% men and 44% women.* | **-** |
